# Rodent models in neuroscience research: is it a rat race?

**DOI:** 10.1242/dmm.026120

**Published:** 2016-10-01

**Authors:** Bart Ellenbroek, Jiun Youn

**Affiliations:** School of Psychology, Victoria University of Wellington, PO Box 600, Wellington 6041, New Zealand

**Keywords:** Addiction, Animal models, Mice, Neuroscience, Rat, Social behaviour

## Abstract

Rodents (especially *Mus musculus* and *Rattus norvegicus*) have been the most widely used models in biomedical research for many years. A notable shift has taken place over the last two decades, with mice taking a more and more prominent role in biomedical science compared to rats. This shift was primarily instigated by the availability of a much larger genetic toolbox for mice, particularly embryonic-stem-cell-based targeting technology for gene disruption. With the recent emergence of tools for altering the rat genome, notably genome-editing technologies, the technological gap between the two organisms is closing, and it is becoming more important to consider the physiological, anatomical, biochemical and pharmacological differences between rats and mice when choosing the right model system for a specific biological question. The aim of this short review and accompanying poster is to highlight some of the most important differences, and to discuss their impact on studies of human diseases, with a special focus on neuropsychiatric disorders.

## Abstract (precis)

**Summary:** In this At a Glance poster review, Ellenbroek and Youn describe important differences between rats and mice that impact on their use as model organisms for brain disorders.

## Introduction

Rats and mice have been the leading model organisms used in biomedical research for well over a century, although other animals, such as non-human primates, zebrafish, fruit flies and roundworms, are also used. However, in recent years a shift in rodent-based research has taken place, with mice rapidly overtaking rats as the major model of choice in biological research. Accordingly, there has been a shift in the proportion of neuroscience-related research using mice from about 20% in the 1970s and 1980s to around 50% in recent years (see poster, panel 1). This shift in preference is likely to be related to the availability of techniques for genetic manipulation of mice, and temporally coincides with the publication of the first knockout mouse in 1987 (Thomas and Capecchi, 1987). Although lagging behind that of mice (see poster, panel 2), the genetic toolbox for rats is now rapidly filling up, with the introduction of N-ethyl-N-nitrosourea (ENU) mutagenesis (Zan et al., 2003), zinc-finger nucleases (Geurts et al., 2010), homologous recombination (Tong et al., 2011) and, most recently, clustered regularly interspaced short palindromic repeats (CRISPR)/Cas-mediated genome editing (Shao et al., 2014). Together with the elucidation of the complete genome of the rat more than a decade ago (Gibbs et al., 2004) and ongoing functional annotation of the genome (data are collected, consolidated and integrated at the Rat Genome Database; see Shimoyama et al., 2016), these advances in technology will likely lead to a rapid increase in genetic rat models as well. This leads to the inevitable question of whether there is a purpose in generating rat models where mouse models are already available. If these two rodents are more or less identical, it would be pointless, and unethical, to replicate the findings in one organism in the other without a valid reason. However, based on a large body of evidence, rats are not simply ‘big mice’ and, although similar in many aspects, there are fundamental differences between these rodents. Indeed, it has long been recognized that, especially in neuroscience and behavioural research, rats have a number of clear advantages, such as the relatively large size of their brains, which makes brain surgery much easier (see poster, panel 3). Rats are also much easier to handle than mice and less easily stressed by human contact. In fact, repeatedly handling rats prior to a behavioural experiment is a routine practice, whereas this procedure in mice often induces more stress in the animal (Meijer et al., 2007). In this At a Glance poster review, we will highlight the major differences between mice and rats that could impact on neuroscience research. We begin by discussing the evolution of these organisms, and then detail some basic differences between rats and mice. We then discuss how these differences impact on technical considerations, social, addictive, impulsive and cognitive behaviours, and studies of neurodegenerative disorders. Overall, we aim to show that rats and mice both have an important role to play in understanding the aetiology, pathophysiology and pharmacology of neuropsychiatric diseases, and that a careful examination of both organisms is necessary before a choice of model for a translational study can be made.

**Figure:**
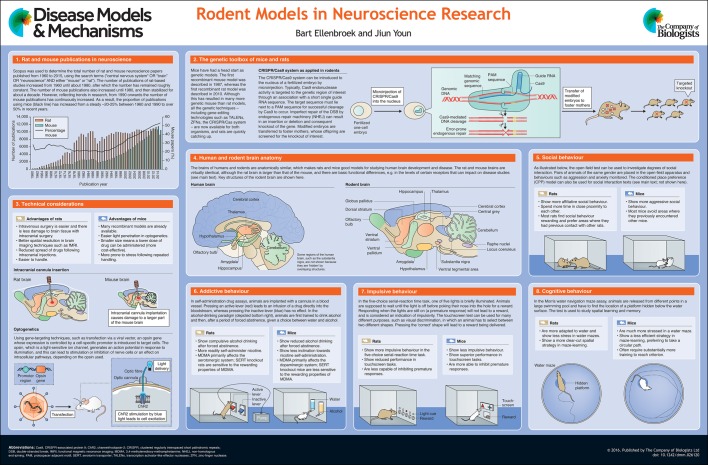
A high-resolution version of the poster is available for downloading at http://dmm.biologists.org/lookup/doi/10.1242/dmm.026120.supplemental.

## Evolutionary divergence of rats and mice

Rats and mice belong to the order Rodentia, which, with over 2200 species, is by far the largest order within the class Mammalia (Wilson and Reeder, 2005). Within the Rodentia, the Muridae constitutes the largest family, with about 700 species, including the Old World rats and mice (rodents found in Eurasia, Africa or Australia, grouped together in the sub-family of the Murinae) (Wilson and Reeder, 2005). Of these, strains derived from *Mus musculus* and *Rattus norvegicus* are used in the overwhelming majority of animal research for biomedical purposes. Thus, in the remainder of this article, when we refer to ‘species differences’, we refer specifically to differences between these two species.

The degree of evolutionary divergence between rats and mice has been hotly debated, especially because estimates of the most recent common ancestor (MRCA) originally led to rather different results depending on the methodology used. Paleontological studies indicated that the MRCA existed 15- to 20-million years ago (mya) (Jaeger et al., 1986), whereas estimates of 27 and even 42 mya were given based on molecular clock data (Huchon et al., 2000; Kumar and Hedges, 1998). The molecular clock technique uses the mutation rate of DNA to deduce when two species have diverged and therefore assumes that mutation rates are constant. However, it has been suggested that murids have higher than normal mutation rates, which would lead to an apparent ‘slowing’ of the molecular clock (because mutations occur faster than in other species). Using complex modelling, and taking the different mutational rates of rats and mice into account, more recent studies estimate the MRCA of rats and mice to have lived about 15-20 mya (Adkins et al., 2001; Steppan et al., 2004) (in line with paleontological data), roughly in the same range as the separation between the macaque monkeys and the great apes, including *Homo sapiens* (Zhou et al., 2014).

It follows that the genetic distance between rats and mice is quite substantial and, consequently, it can be assumed that the functional differences, both at the molecular and behavioural level, are equally large. Differential expression of genes in the mouse and rat brains was recently investigated in two of the most often used rodent models (especially in the neuroscience field), namely the Sprague Dawley rat and C57BL/6 mice (Francis et al., 2014). Using microarray-based analysis, the authors found that 4713 out of a total of 10,833 genes were differentially expressed in the dendrites of hippocampal neurons between rats and mice. By comparison, only 54 genes were differentially expressed between two different, often-used, mouse strains (C57BL/6 and Balb/c). Considering the importance of the hippocampus in behaviour (especially memory), this finding is relevant in understanding the species differences in many cognitive tests, as discussed below. Intriguingly, the authors also compared other tissues (including heart, skeletal muscle, intestines etc.) between rats and mice, and, although differences in these tissues were also substantial, they were much less dramatic than those found in the hippocampus.

Given the considerable transcriptomic variation between rats and mice, it is not surprising that multiple differences in the general biology of these organisms have been described. It would be beyond the scope of this review to describe all of these. Instead, we will focus below on describing several important differences that can (a) have a bearing on experimental considerations when making a choice of model, and (b) impact on responses and phenotypes in the study of human neurobiological dysfunction.

## Basic differences between mice and rats: technical considerations

One of the most obvious differences between rats and mice is in size and weight, with rats weighing roughly about eight to ten times more than mice in adulthood. The greater size of rats provides a number of practical advantages, especially in relation to surgical procedures and in studies of spinal cord injury, where rat models have been of great translational value (reviewed in Kjell and Olson, 2016). An often-used technique in addiction research involves the implantation of a catheter in a blood vessel, to allow for drug self-administration, and this is more readily achieved in the larger animal (Feduccia and Duvauchelle, 2010). Another frequently used technique is intracerebral cannula implantation, in which a small cannula is implanted into the brain (see poster, panel 3) (Kokare et al., 2011). This can be used to locally administer a drug directly into a specific brain region, allowing the role of this brain region in a behavioural phenotype to be examined. This technique can also be used to implant a microdialysis probe to enable sampling of extracellular fluid in the brain, which can be useful for measuring neurotransmitter concentrations. The larger size of the rat brain not only makes surgery easier, but the cannula tends to cause less damage and a smaller region is affected, increasing the sensitivity of the method. Finally, more and more techniques for brain imaging in animals are being developed, based on functional magnetic resonance imaging (fMRI) (Bartelle et al., 2016), positron emission tomography (PET) (Zimmer et al., 2014) or near-infrared brain imaging (Kim et al., 2014). In all of these techniques, the larger brain of the rat offers better spatial resolution. In addition, it has been shown that rats can be trained to sit still during such imaging procedures, thus cancelling the need for anaesthesia, which interferes with normal brain activity (Febo, 2011). This has not been shown to be possible in mice (Harris et al., 2015).

That being said, it should also be noted that the smaller size of mice can be an advantage for certain techniques, such as in optogenetics (see poster, panel 3). This method is used for precise stimulation or inhibition of neuronal pathways in freely moving animals, and is based on the transfection of a specific set of neurons with specific light-sensitive proteins that can subsequently be activated by illumination (Deisseroth, 2015). Using a gene-targeting approach, a specific channelrhodopsin (light-sensitive ion channels) is introduced to target cells. Because the expression of these channels is under the influence of cell-specific promoters, only certain cells will express the channelrhodopsin and hence be sensitive to light. Depending on the type of channelrhodopsin used, illumination can have different effects. For instance, channelrhodopsin-2 (ChR2), a blue-light-sensitive cation channel, leads to excitation of the cells, whereas halorhodopsin (NpHR), a yellow-light-activated chloride pump, will induce inhibition. Recently, more advanced channelrhodopsins have been designed, such as opsin-receptor chimaeras (OptoXR), a rhodopsin-GPCR (G protein-coupled receptor) chimera that responds to green light stimulation. Stimulation of OptoXR leads to more complex intracellular changes, such as increases in Ca^2+^ or cyclic AMP, which lead to changes in signalling pathways. The smaller brain of mice makes it easier for light to pass through and reach deeper brain regions; thus, optogenetics is currently more commonly used in mice. Likewise, the smaller weight of mice is a distinct advantage in drug development, because novel compounds – which are usually costly and only available in very small quantities – are typically given in doses relative to the body weight of the animal.

## Basic functional differences between mouse and rat brains

Although the rat and mouse brains are anatomically identical, several major functional differences have been described. For instance, fundamental species differences in the distribution of a subtype of serotonin (5-HT) receptor, 5-HT6, have been reported (Hirst et al., 2003). Serotonin is a neurotransmitter involved primarily in emotional regulation, cognition and impulsivity, and changes in serotonin functioning have been implicated in a variety of disorders, most prominently mood disorders (Haase and Brown, 2015), anxiety disorders (Corchs et al., 2015) and autism spectrum disorders (ASDs) (Gabriele et al., 2014). Serotonin interacts with multiple receptors, with the 5-HT6 receptor currently being investigated in relation to depression, Alzheimer's disease (AD), dementia and drug addiction (Karila et al., 2015). Rats, like humans, demonstrate very high levels of *5-HT6* mRNA and receptor-binding in the dorsal and ventral striatum, which are subcortical brain structures (see poster, panel 4) that play a key role in reward perception, habit formation, cognitive flexibility and motor functioning, and which are involved in most psychiatric disorders, including schizophrenia, drug addiction and attention deficit hyperactivity disorders (ADHD). By contrast, 5-HT6 receptor levels in the same regions in the mouse brain are very low. In addition, pharmacological differences resulting from changes in the binding pocket of the 5-HT6 receptor have been found. More specifically, residues 188 (tyrosine in mice; phenylalanine in rats and humans) and 290 (serine in mice; asparagine in rats and humans) were identified as key amino acids for binding selective ligands. As a result, whereas certain antagonists bind with high affinity to human and rat 5-HT6 receptors, they have much lower affinity for the mouse 5-HT6 receptor. This implies that the mouse is less suitable for identifying drugs with high affinity for the human 5-HT6 receptor, making the rat a preferred model for drug development in this area of research.

Differences have also been reported in the expression of LRRK2 (leucine rich repeat kinase 2), a neuronal protein encoded by the *PARK8* gene (Klein and Westenberger, 2012). Whereas, in mice, LRRK2 has a relatively widespread distribution, in rats the distribution is much more restricted (West et al., 2014). Of particular note, high levels of LRRK2 have been detected in the dopaminergic cell body region of the substantia nigra pars compacta in mice, but not in rats. *PARK8* has been linked to Parkinson's disease (PD) (Klein and Westenberger, 2012), suggesting that these differences could impact on studies of this neurodegenerative disease (discussed further below).

Another example of a key functional difference between the rat and mouse brains is provided by kisspeptin, a GPCR with a role in the release of gonadotropin-releasing hormone (GnRH) via the hypothalamus-pituitary-gonadal axis. The distribution of kisspeptin-positive cells within the hypothalamus differs between the two species (Overgaard et al., 2013). Whereas high levels of these cells are found in the mouse periventricular nucleus (a structure located in the hypothalamus, which plays a key role in the release of hormones from the pituitary gland), levels in the rats are much lower. Intriguingly, mRNA levels are thought to be similar between the two species, suggesting that the difference is mediated at the post-translational level. Recently, kisspeptin was implicated in schizophrenia using the maternal immune-activation mouse model, a well-established model in which pregnant animals are injected with an immunogenic substance (such as polyl:C) to simulate a viral infection and trigger neurodevelopmental abnormalities in the fetus. In this study, the offspring displayed a variety of schizophrenia-like abnormalities (Meyer et al., 2009), which correlated with a significant reduction in kisspeptin levels (Cardon et al., 2010). So far, the functional importance of kisspeptin in schizophrenia is virtually unknown, and the differential expression of the protein in rat and mouse brains thus warrants further investigation.

Finally, there are important and fundamental differences between rats and mice with respect to neurogenesis (birth of new neurons) within, among other regions, the hippocampus. The hippocampus is one of the few brain regions where, even in adulthood, new neurons continue to be formed, and this process is thought to be involved in learning and memory (Lazarov and Hollands, 2016). Moreover, there is compelling evidence that neurogenesis is reduced in the context of depression and normalized after chronic antidepressant treatment (Miller and Hen, 2015). In a recent study, it was shown that the rate of neurogenesis in the adult hippocampus was much larger in rats than in mice. More importantly, in rats, these new cells matured about 2 weeks earlier, were twice as likely to escape cell death and were ten times more likely to be activated during learning than in mice (Snyder et al., 2009). Thus, this study revealed substantial differences in neuronal plasticity between rats and mice, which was not limited to the hippocampus but extended into the cortical regions as well.

Overall, a wealth of studies has delineated several key differences in functional anatomy, physiology and pharmacology between rats and mice. Given that these differences are found predominantly in two major communicative systems in the body – the nervous and the endocrine systems – it is not surprising that rats and mice show fundamentally different responses in studies of human diseases, particularly behavioural and neuropsychiatric disorders.

## Rats and mice as models for human neurological and psychiatric disorders

The most important reason for using rats and mice in research is to model aspects of human physiology and function, most notably to advance our understanding of human diseases. Rodent models have proven invaluable for the development of new drugs for many human conditions, although it should equally be recognized that the translational value of all model systems, especially within the field of neuroscience, is still far from perfect. In the remainder of this article, we will highlight some important differences between rats and mice in relation to specific neurological disorders modelled in both intact and genetically altered animals.

### Differences in social behaviour

Social cognition can be defined as all processes that are elicited by and/or directed towards other subjects, and deficits in social cognition are a feature of a variety of disorders, including schizophrenia, ASD, ADHD and bipolar disorder. Hence, the study of deficits in social cognition is pertinent in the study of these diseases, especially ASD, for which a large number of genetic models have been developed. A relatively recent analysis showed that at least 70 different genetic mouse models have been investigated in relation to ASD (Banerjee-Basu and Packer, 2010). Given the relatively large genetic toolbox for mice, this is not surprising. However, there are features of rats that make them an attractive alternative model for ASD. Indeed, substantial differences in social cognition and behaviour have been described between rats and mice, and these are likely related to differences in the natural social structure of both species. Although both rats and mice live in large hierarchical groups, rats are much less territorial and the hierarchy between males is far from absolute. In line with this, it is quite common for all males to mate with all females, although the dominant male will have the largest number of intromissions and ejaculations (Mcclintock and Anisko, 1982; Mcclintock et al., 1982). It is thought that this behaviour reduces aggression and infanticide – the presence of males in litters in laboratory settings has even been found to enhance growth and survival of the offspring (Lore and Flannelly, 1977). Blanchard et al. (2001) further demonstrated that aggression is relatively low in rats. In their study, the authors used an innovative visual burrow system to mimic the natural environment of the rat and populated it with male and female animals. Analysis of rat behaviour showed that aggression between dominant and subordinate males was negligible after the first few hours of colony formation. In contrast, mice live in more territorial structures (called ‘demes’) founded by a single male that mates with multiple females (van Zegeren, 1980). Mice burrows are thus much less complex than rat burrows, and usually only have a single cavity occupied by a single male (Schmid-Holmes et al., 2001). As a result, interactions between males are much rarer and, when they occur, are more aggressive and territorial in nature.

These differences in the natural social structure of mice and rats also affect the social behaviours they display in the laboratory setting. Casual investigations in the home cage reveal that mice show much more aggressive behaviour towards conspecifics than rats do. Likewise, in an ‘open-field’ experiment looking at dyadic interactions (two animals are placed in a large enclosure that is new to them; see poster, panel 5), it was shown that the average distance between two rats is smaller than between two mice, and, related to this, the latency to start interacting is shorter and the total frequency of interactions higher in rats (Vestal, 1977). These findings were recently confirmed and extended using a conditioned place preference (CPP) model (Kummer et al., 2014; Zernig and Pinheiro, 2015). CPP is a well-validated model for assessing the rewarding or aversive properties of drugs of abuse or other rewarding stimuli such as social interaction (Tzschentke, 2007). Using social interaction as the rewarding stimulus, animals are first given free access to a box with two different compartments and their preference for either chamber is measured. During the next phase they are repeatedly placed in the less-preferred compartment with another animal and in the preferred compartment alone. After this conditioning phase, the animals are given free access again to both compartments. An increase in time spent in the less-preferred (social-stimulus-paired) compartment is taken as an indication of the rewarding property of social interaction. Zernig and colleagues showed, that during a 15 min encounter, rats engaged in social interaction for 79% of the time, whereas mice spent only 22% of their time interacting with each other (Kummer et al., 2014). Consistent with this, the authors found that, whereas 85% of the rats found the interaction rewarding (i.e. the rats spent more time in the social-stimulus-paired compartment), only half of the mice found it rewarding and half of them actually found it aversive (i.e. the mice spent less time in the social-stimulus-paired compartment). Moreover, rats found social interaction as rewarding as 15 mg/kg body weight cocaine, whereas mice found this dose of cocaine much more rewarding than social interaction. In summary, whereas the majority of rats readily engage in social behaviour and find it rewarding, mice spend significantly less time interacting with a conspecific and many even find it aversive.

This conclusion is supported by data obtained using a completely different approach. In an attempt to understand the impact of communal nesting, the offspring of animals where the mother reared the pups alone or in groups of four females in a larger communal nesting area were compared. Whereas, in mice, communal rearing actually led to an increase in anxiety in adulthood, in rats, the opposite effect was seen. Rats reared communally showed significantly reduced levels of anxiety (Branchi and Alleva, 2006; Branchi et al., 2006; Martinez et al., 2015). Anxiety in these studies was measured using either an open-field or ‘elevated plus’ maze, both of which are tests assessing the conflict between exploration of novel environments and fear of open spaces. In the open-field test for anxiety, animals are placed in a relatively large box with walls, and the percentage of time spent in the (exposed) centre of the open field is compared to the time spent along the (safe) area near to the walls is taken as an indication of the level of anxiety. The elevated plus maze consists of four arms forming a cross, elevated above the ground. Two of the arms have relatively high walls (closed arms), whereas the remaining two arms do not have walls (open arms). The percentage of time spent in the open versus closed arms is taken as an indication of the level of anxiety experienced by the animals (Pellow et al., 1985).

Considering the complex and collaborative nature of human social behaviour, these species differences are highly relevant in modelling social behaviour deficits. Particularly in relation to ASD, where reductions in social behaviour and social communication are core symptoms, mice might not be the ideal model given their intrinsic lack of receptiveness to social interaction (Moy et al., 2008, 2007). Several rat models for ASD have been developed recently, primarily based on environmental manipulations such as maternal immune activation or prenatal exposure to valproate (Patterson, 2011). The development of specific genetic rat models is an important step towards understanding this, and related, psychiatric disorders.

### Differences in addictive behaviour

Some fundamental differences in addictive behaviours between rats and mice have also been identified (see poster, panel 6). In a typical rodent self-administration drug assay, animals are usually given a choice between two responses (such as a left and right lever press). Whereas one response is without consequences (e.g. pressing the inactive lever), the other response (active lever) will lead to the injection of a small quantity of drug directly into the bloodstream of the animals, via a cannula. A drug with addictive properties, such as cocaine, methamphetamine and heroin, will usually lead to a rapid increase in active lever presses in both rats and mice (Moser et al., 2011). However, rats, like humans, show a strong alcohol-deprivation effect such as a compulsive drinking pattern after repeated periods of deprivation; an effect that has not been demonstrated in mice (Vengeliene et al., 2014). Another study reported a clear difference between rats and mice in terms of the effects of cannabinoid CB2 receptors on the rewarding properties of cocaine (Zhang et al., 2015). The authors found that CB2 agonists increase the rewarding effects of cocaine in rats but decrease the rewarding effects in mice. Whether this is due to a fundamental difference in receptor localization awaits experimental confirmation, but, compared to the rat, both the cell body region of the ventral tegmental area (see poster, panel 4) as well as the terminal regions (i.e. the striatum), the nucleus accumbens and prefrontal cortex of mice display a substantially higher density of CB2 receptors (Zhang et al., 2015). So far, CB2 agonists have not been evaluated in humans with drug addiction and it is therefore premature to conclude whether rats or mice more closely resemble humans in their responses.

Differences have also been reported in relation to the rewarding properties of nicotine. Compared to other addictive drugs (such as cocaine, methamphetamine or heroin), nicotine is substantially less rewarding/addictive, making it more challenging to self-administer in animals. Despite the challenges, nicotine self-administration has routinely been reported in rats (Brennan et al., 2015). Mice, on the other hand, do not readily self-administer nicotine (Parker et al., 2014). For instance, Contet and colleagues found that, although mice would selectively press the active nicotine-paired lever, there was no dose-response effect and turning on the associated light cue alone was sufficient to maintain responding on the lever, thus questioning the relative contribution of nicotine in lever pressing (Contet et al., 2010).

Fundamental differences between rats and mice have also been found with respect to rewarding properties of MDMA (3,4-methylenedioxy-methamphetamine), the active ingredient of ecstasy. MDMA increases serotonin levels in the brain, mainly by inhibition of serotonin-reuptake transporters. In mice, genetic deletion of the serotonin transporter (SERT) was shown to result in decreased sensitivity to the rewarding effects of MDMA (Trigo et al., 2007). By contrast, rats with the same genetic deletion of the serotonin transporter were significantly more sensitive to the rewarding properties of MDMA (Oakly et al., 2014). Interestingly, several other differences between the species in relation to the behavioural and biochemical effects of MDMA have been reported (Easton and Marsden, 2006), with some data suggesting that the effects are mediated mostly via dopamine in mice but predominantly via serotonin in rats (Kindlundh-Hogberg et al., 2007) and humans (Roberts et al., 2016). Although the relationship between serotonin-transporter polymorphisms and the reinforcing effects of MDMA have not yet been studied in humans, there is evidence that some of the characteristic behavioural effects are more prominent in humans with a genetic reduction in the serotonin transporter, similar to what has been described in rats (Pardo-Lozano et al., 2012).

Together with several other differences between rats and mice in processes that are fundamental to addictive behaviour [such as differences in impulsivity (see below)], this suggests that rats are the optimum rodent model for studies of human addictive behaviour (Parker et al., 2014).

### Differences in impulsive behaviour

Impulsivity – the tendency to act before thinking – is thought to consist of several different components, most notably motor and cognitive impulsivity. Motor impulsivity (also referred to as impulsive actions) refers to the inability to withhold a motor response, whereas cognitive impulsivity (impulsive choice) refers to the inability to choose a delayed large reward over an immediate small reward. There is ample evidence that both forms of impulsivity are linked to the propensity to develop drug addiction (Grant and Chamberlain, 2014; Jupp et al., 2013). One of the most often used paradigms for measuring motor impulsivity is the five-choice serial-reaction time task (see poster, panel 7). In this task, animals are placed in a box with five holes, each with a stimulus light above it (Bari and Robbins, 2013). One of the five stimulus lights is briefly turned on and the animal is required to make a response (nose poke in the correct hole) to obtain a food reward. A crucial element of the task is that the subject has to withhold its response until the light has been switched off. Premature responding, i.e. making a nose poke while the light is still on, is seen as a sign of motor impulsivity. Studies comparing rats and mice have shown that rats are, in general, more impulsive in their actions and seem less able to inhibit premature responding (Young et al., 2013). It has been suggested that this increased impulsivity also affects other aspects of learning, such as touch-screen learning, where rats typically need an additional barrier to prevent too-fast (inappropriate) responding (Young et al., 2013). In such tests, animals have to use their nose to touch a specific image projected onto a touchscreen, in contrast to traditional operant chambers where animals generally need to press a lever. On the other hand, cognitive impulsivity is often measured using the delay discounting task (Bari and Robbins, 2013). Although there are different versions of this assay, it usually involves altering the delay between a ‘high’ and ‘low’ reward (for instance, four vs two sucrose pellets, respectively) and establishing the so-called ‘indifference’ point in which animals are equally likely to choose either reward. Studies have shown that, whereas rats can learn the delay discounting task fairly well, mice demonstrate substantially more difficulty; it takes significantly longer to train mice, and their response is much more variable than that in rats (Parker et al., 2014).

### Differences in cognition

Cognitive deficits are a core feature of many psychiatric and neurological disorders, most prominently dementia and schizophrenia. However, cognition is a very broad concept and encompasses a large number of different components, such as short- and long-term memory. One of the most often used paradigms in animal research for assessing spatial learning and memory (i.e. learning to locate an object in relation to its surrounding) is the Morris water maze. In this assay, animals are placed in a circular pool filled with water in which an invisible platform is submerged under the surface (see poster, panel 8). By placing the animals in different starting positions within the pool, they are trained to find the hidden platform based on external cues. It has been shown that, compared to rats, mice have greater difficulty in learning to find the platform (Whishaw and Tomie, 1996). It has been suggested that this is to a large degree related to fundamental species differences, i.e. in the wild, rats build burrows near water and spend a considerable time swimming, whereas mice tend to avoid water when possible. Indeed, in an analogous study conducted in a ‘dry maze’, no differences between rats and mice were found (Whishaw and Tomie, 1996). A more detailed analysis showed that the performance of mice is to a large degree related to ‘non-cognitive’ effects (Lipp and Wolfer, 1998). Using a factor analytical approach, the authors found that the major contributor to learning performance in mice is the degree of thigmotaxis (i.e. to what extent mice swim around the outside walls of the pool) as opposed to the development of an actual spatial learning strategy. It should also be noted that, especially in mice, some strains are able to learn to find the platform more effectively than other strains, indicating that strain-to-strain variation also exists (Vorhees and Williams, 2015). Overall, these studies indicate that, even if mice eventually learn to locate the platform, they use a different strategy from rats, and thus the test may be less appropriate for studying spatial memory in mice than it is for rats.

Other differences in learning between rats and mice exist as well. Although not studied very systematically, mice often need substantially longer habituations and training sessions to perform certain tasks than rats do (Colacicco et al., 2002; Jaramillo and Zador, 2014; Prusky et al., 2000) and, in association with this, experience more stress and anxiety. As an example, Colaccico and colleagues studied mice in a task designed to measure executive functioning (required for higher-level problem solving) (Colacicco et al., 2002). In this test, animals are required to learn the location of a food reward in one of two bowls that differ in two dimensions (usually scent and digging material). For instance, the bowls can be scented with thyme or cinnamon and can contain wood chips or paper strips as digging materials. Only recognition of one specific dimension is rewarded (e.g. only thyme-scented bowls). Once the animals reliably dig in the correct bowl, they are presented with new combinations and have to switch either to another scent (intra-dimensional shift) or to a digging material (extradimensional shift). Given the complexity of the design, animals have to go through a series of trials lasting for several hours. The authors (Colacicco et al., 2002) report that the performance over time became much more erratic in mice than in rats and hence the maximal duration of each trial for mice was set at 1 h, and training had to be spread out over multiple days. Thus, in general, rats are the preferred species for cognitive tests because they perform more stably over time and are less affected by non-cognitive distractors (such as stress, thigmotaxis etc.).

### Models for neurodegenerative diseases

Rats and mice have also been extensively used as model systems for neurodegenerative diseases, including PD, Huntington's disease (HD) and AD. For instance, mice are sensitive to the dopaminergic neurotoxin MPTP (1-methyl-4-phenyl-1,2,3,6-tetrahydropyridine), whereas rats are relatively resistant to its toxic effects (Bové and Perier, 2012). Although this has implications for modelling PD in rats and mice, it is important to note that this is more related to rate of conversion of MPTP into the active metabolite MPP^+^ (1-methyl-4-phenylpyridinium) rather than a fundamental difference in the dopaminergic system. Indeed, MPP^+^ is effective as a neurotoxin in both species (Bové and Perier, 2012). Another available rat model for PD is based on genetic modification of α-synuclein, a presynaptic neuronal protein with putative roles in synaptic plasticity and dopamine release (Lashuel et al., 2013). Consistent with the role of α-synuclein in PD pathology, rare point mutations in its gene, *SNCA*, are implicated in familial forms of the disease (Belin and Westerlund, 2008). Intriguingly, although overexpression of mutated *SNCA* induces severe dopamine loss in the rat, a similar effect has not been reported by overexpressing the gene in mice (Nuber et al., 2013). In line with the dopaminergic cell loss, rats develop clear deficits in motor behaviour that are reminiscent of PD.

Differences between rats and mice have also been described in relation to modelling HD, with some studies suggesting that the rat provides a more faithful model of the human disease than the mouse (von Horsten et al., 2003), especially in terms of progression, which seems much faster in mice than in rats (and humans). However, this conclusion is mainly based on a comparison with the R6/2 mouse model, which shows a very fast-progressing and severe phenotype (Mangiarini et al., 1996), even compared to other mouse models. Many genetic mouse and rat models of HD have now been developed, each with unique characteristics (Abada and Ellenbroek, 2016). We recently compared the BAC-HD rat and mouse models, in which the same genetic construct (i.e. the full-length human *HTT* gene, encoding huntingtin) is inserted using a bacterial artificial construct (BAC). Although there are many similarities between the two models, particularly in relation to motor symptoms – such as a reduction in spontaneous motor activity and motor coordination – clear differences were also seen (Abada et al., 2013b,c). For instance, deficits in sensorimotor gating (prepulse inhibition) were reported in BAC-HD mice (Menalled et al., 2009) but not in rats (Abada et al., 2013b). Prepulse inhibition refers to the inhibitory effect of a weak stimulus on the acoustic startle response evoked by a loud noise, and this response has been found to be reduced in individuals with HD (Swerdlow et al., 1995). Even more pronounced differences were found in terms of conditioning of fear. A typical test involves presenting animals with a stimulus before giving them a brief, mild electric shock. The next day, the animals are placed in the same cage and tested for the onset of ‘fear conditioning’, recorded as an increase in freezing posture, either by being confronted with the same environmental context in which they received the shock, or after turning on a stimulus (i.e. a tone or light) that was previously associated with the onset of the shock. Interestingly, whereas fear conditioning is enhanced in BAC-HD mice (Abada et al., 2013c), it is reduced in BAC-HD rats (Abada et al., 2013a). It is currently unclear how these deficits in associative memory could be relevant in human HD; however, these studies demonstrate the importance of carefully characterizing and comparing rodent models before using them to address a specific question in neuroscience.

Finally, both rat and mouse models have been developed for AD. AD is the most common cause of dementia and leads to substantial deficits in memory accompanied by the formation of specific pathological bodies in the brain, notably plaques and tangles (Mattson, 2004). Although the aetiology of AD is complex and far from understood, the identification of several (albeit relatively rare) mutations in genes such as *APP* (which encodes amyloid precursor protein) and *PS1* and *PS2* (which encode presenilin 1 and 2, which are involved in the metabolism of APP) has led to the overarching ‘amyloid’ hypothesis, which states that overexpression of β-amyloid is involved in the pathology of AD. Many transgenic animal models for AD have been developed on the basis of these genetic associations and the amyloid hypothesis. Again, the majority of models that have so far been studied are mouse models, but several rat models have been developed in recent years. This is important because, in addition to the aforementioned benefits of rats over mice in cognitive tests, rats, like humans, have six isoforms of the tau protein (Hanes et al., 2009). Hyperphosphorylation of tau is involved in the formation of tangles, an essential pathological hallmark of AD, and the similarity in isoforms between humans and rats could indicate a higher degree of similarity in tangle formation as well. A key challenge that has emerged in using rats to model AD is that transgenic rats do not seem to develop AD-like plaques. For instance, the Tg6590 rat model (based on a mutation observed in Swedish cohorts of AD) does not display mature plaques (Kloskowska et al., 2010), whereas the mouse equivalent (Tg2576) shows substantial plaque formation (Hsiao et al., 1996). Intriguingly, cognitive deficits are present in both rat and mouse models, calling into question the causal relationship between plaques and dementia. Other phenotypical differences have also been found between transgenic rat and mouse models of AD. For instance, the TgF344-AD rat model, which is also based on a patient-specific mutation, showed a much higher abundance of soluble oligomeric β-amyloid structures, as well as tangle-like pathologies and neuronal cell loss, than the comparable mouse model (Do Carmo and Cuello, 2013). It is currently unclear whether these differences are due to unrelated factors, such as differential rates of transfection or gene expression, or whether they represent fundamental species differences in APP processing and functioning (Do Carmo and Cuello, 2013).

## Conclusions

Although rats and mice belong to the same family and obviously share many important features, there are several fundamental differences that impact on the choice of model in disease studies, particularly in neuroscience. The differences described here mostly refer to those reported when comparing the most often used rat (Wistar and Sprague Dawley) and mouse (C57BL/6 and Balb/c) strains. However, studies comparing different mouse (Bohlen et al., 2014; Moy et al., 2008) or rat (Clemens et al., 2014; Gomes et al., 2013) strains have also identified substantial differences within, as well as between, individual species, indicating that the full extent of variation between rats and mice might yet be underappreciated.

The increased availability of genetic tools for the rat, including zinc-finger nucleases, transposons, transcription activator-like effector nucleases (TALENs), CRISPR/Cas9 (see poster, panel 3) and more efficient pluripotent embryonic-stem-cell based technologies, are likely to pave the way for a multitude of new genetic models based in the rat. This is an important development because the vast majority of neuroscience research was originally done using rats (see poster, panel 1), and substantial adaptations to early rat-based paradigms were needed to accommodate the subsequent dominant use of mice. Evidently, such adaptations involve much more than just reducing the size of the cage, and basic differences in social behaviour, fear of water and impulsivity need to be taken into account before a ‘rat task’ can be successfully adapted to mice. Indeed, it has even been suggested that rats seem to be superior models for human neuroscience simply because the behavioural tests predominantly used were originally perfected for this animal (Timberlake, 2001, 2002). The development of a novel genetic rat model obsoletes the necessity to alter the protocols and designs to fit mice. Of course, this does not imply that mice do not have a place in neuroscience research, but that mice need not be used for behavioural experiments that they are not well-adapted for on the basis of technical considerations. As we have discussed, rats and mice can show diametrically opposite responses in basic studies of cognition, addiction, impulsive and social behaviour, and demonstrate differences in extent of neurodegeneration, demonstrating the importance of choosing the ‘best’ model for a human condition wisely. The translational validity of current animal models for brain diseases has been relatively poor to date (Ellenbroek and Youn, 2016; Kaffman and Krystal, 2012), and there is an urgent need for improvement. Although the usefulness of developing transgenic rats has sometimes been questioned (“we already have transgenic mice” is commonly heard), we believe that the further development of these models is of crucial importance and far from a ‘rat race’.
